# Effect of a polygenic risk score in patients with late-onset, early-onset, familial, or hereditary colorectal cancer

**DOI:** 10.1093/jncics/pkag041

**Published:** 2026-04-11

**Authors:** Hannah Klinkhammer, Isabel Spier, Claudia Perne, Per Hoffmann, Reinhard Büttner, Evelin Schröck, Silke Redler, Arne Jahn, Marcus Franke, Gabriela Möslein, Matthias Kloor, Börge Schmidt, Deepak Vangala, Huu Phuc Nguyen, Andreas J Forstner, Verena Steinke-Lange, Elke Holinski-Feder, Robert Hüneburg, Carlo Maj, Christoph Engel, Andreas Mayr, Stefan Aretz

**Affiliations:** Institute for Medical Biometry and Statistics, Philipps-University of Marburg, Marburg, Germany; Institute for Genomic Statistics and Bioinformatics, University Hospital Bonn, Bonn, Germany; Institute of Human Genetics, Medical Faculty, University of Bonn, Bonn, Germany; National Center for Hereditary Tumour Syndromes, University Hospital Bonn, Bonn, Germany; Hereditary Cancer Syndrome Center, ERN GENTURIS, Bonn, Germany; Institute of Human Genetics, Medical Faculty, University of Bonn, Bonn, Germany; Institute of Human Genetics, Medical Faculty, University of Bonn, Bonn, Germany; Institute of Pathology, Cologne University Hospital, Cologne, Germany; Institute for Clinical Genetics, University Hospital Carl Gustav Carus at TUD Dresden University of Technology and Faculty of Medicine of TUD Dresden University of Technology, Dresden, Germany; Hereditary Cancer Syndrome Center, ERN GENTURIS, Dresden, Germany; National Center for Tumor Diseases (NCT), NCT/UCC Dresden, a partnership between DKFZ, Faculty of Medicine and University Hospital Carl Gustav Carus, TUD Dresden University of Technology, and Helmholtz-Zentrum Dresden-Rossendorf (HZDR), Dresden, Germany; German Cancer Consortium (DKTK), Dresden, Germany; German Cancer Research Center (DKFZ), Heidelberg, Germany; Institute of Human Genetics, Medical Faculty, University Hospital Düsseldorf, Heinrich-Heine-University, Düsseldorf, Germany; Institute for Clinical Genetics, University Hospital Carl Gustav Carus at TUD Dresden University of Technology and Faculty of Medicine of TUD Dresden University of Technology, Dresden, Germany; Hereditary Cancer Syndrome Center, ERN GENTURIS, Dresden, Germany; National Center for Tumor Diseases (NCT), NCT/UCC Dresden, a partnership between DKFZ, Faculty of Medicine and University Hospital Carl Gustav Carus, TUD Dresden University of Technology, and Helmholtz-Zentrum Dresden-Rossendorf (HZDR), Dresden, Germany; German Cancer Consortium (DKTK), Dresden, Germany; German Cancer Research Center (DKFZ), Heidelberg, Germany; Institute for Clinical Genetics, University Hospital Carl Gustav Carus at TUD Dresden University of Technology and Faculty of Medicine of TUD Dresden University of Technology, Dresden, Germany; Hereditary Cancer Syndrome Center, ERN GENTURIS, Dresden, Germany; National Center for Tumor Diseases (NCT), NCT/UCC Dresden, a partnership between DKFZ, Faculty of Medicine and University Hospital Carl Gustav Carus, TUD Dresden University of Technology, and Helmholtz-Zentrum Dresden-Rossendorf (HZDR), Dresden, Germany; German Cancer Consortium (DKTK), Dresden, Germany; German Cancer Research Center (DKFZ), Heidelberg, Germany; Surgical Center for Hereditary Tumors, BETHESDA Hospital Duisburg, Academic Hospital University of Düsseldorf, Duisburg, Germany; Department of Applied Tumor Biology, Heidelberg University Hospital, Heidelberg, Germany; Institute for Medical Informatics, Biometry and Epidemiology, University Hospital of Essen, University of Duisburg-Essen, Essen, Germany; Outpatient Center for Hematology and Oncology, Cancer Center Bochum-Herne, Bochum, Germany; Department of Human Genetics, Medical Faculty, Ruhr-University Bochum, Bochum, Germany; Department of Human Genetics, Medical Faculty, Ruhr-University Bochum, Bochum, Germany; Institute of Human Genetics, Medical Faculty, University of Bonn, Bonn, Germany; Institute of Neuroscience and Medicine (INM-1), Research Center Jülich, Jülich, Germany; Medizinische Klinik und Poliklinik IV, Campus Innenstadt, Klinikum der Universität München, Munich, Germany; MGZ-Medical Genetics Center, Munich, Germany; Hereditary Cancer Syndrome Center, ERN GENTURIS, Munich, Germany; Medizinische Klinik und Poliklinik IV, Campus Innenstadt, Klinikum der Universität München, Munich, Germany; MGZ-Medical Genetics Center, Munich, Germany; Hereditary Cancer Syndrome Center, ERN GENTURIS, Munich, Germany; National Center for Hereditary Tumour Syndromes, University Hospital Bonn, Bonn, Germany; Hereditary Cancer Syndrome Center, ERN GENTURIS, Bonn, Germany; Department of Internal Medicine I, University Hospital Bonn, Bonn, Germany; Centre for Human Genetics, University of Marburg, Marburg, Germany; Institute for Medical Informatics, Statistics and Epidemiology (IMISE), University of Leipzig, Leipzig, Germany; Institute for Medical Biometry and Statistics, Philipps-University of Marburg, Marburg, Germany; Institute of Human Genetics, Medical Faculty, University of Bonn, Bonn, Germany; National Center for Hereditary Tumour Syndromes, University Hospital Bonn, Bonn, Germany; Hereditary Cancer Syndrome Center, ERN GENTURIS, Bonn, Germany

## Abstract

**Objective:**

This study investigates how a polygenic risk score (PRS) influences colorectal cancer (CRC) risk across clinically and molecularly defined risk groups.

**Methods:**

In total, 1839 European-descendant individuals were stratified according to low (<20%), intermediate (20% to 80%), or high (>80%) PRS, based on 93 CRC-associated single-nucleotide polymorphisms (SNPs), for 4 high-risk groups: (i) Lynch syndrome (LS; with CRC: *n* = 679, CRC-free carriers: *n* = 422); (ii) early-onset sporadic CRC (EOS-CRC; *n* = 518); (iii) positive family history for CRC (F-CRC; *n* = 220); and, in EOS-CRC and F-CRC patients, (iv) MSI/dMMR CRC (*n* = 144) vs MSS/pMMR CRC (*n* = 485). CRC risk was compared with population-based controls (*n* = 3119) and late-onset sporadic CRC patients from UK Biobank (LOS-CRC; *n* = 781) using multivariable logistic regression and Cox models.

**Results:**

Polygenic risk score (PRS) was significantly increased in all risk groups compared with population controls. Being in the high PRS category doubled CRC risk in EOS-CRC and F-CRC corresponding to cumulative incidences before 50 and 75 years of 24% and 13%, respectively. Polygenic risk score was significantly higher in EOS-CRC than LOS-CRC. In LS, PRS in non-CRC carriers lay between CRC-LS and population controls. Non-LS individuals with MSI/dMMR tumors showed significantly lower PRS than those with pMMR/MSS tumors, but no difference compared with LS CRC individuals.

**Conclusions:**

Polygenic risk score most strongly influences CRC risk in unexplained EOS- and F-CRC. The effect in LS individuals strongly depends on the penetrance of the altered gene and study design. Nonsignificant trends can partly be explained by the sample size of subgroups. Larger collaborative, prospective studies are needed to validate PRS for personalized CRC risk stratification.

## Introduction

Colorectal cancer (CRC) is the third most incident cancer and the second leading cause of cancer-related death worldwide, with 4 times higher incidence rates in the Global North.^1^ Established CRC risk factors include Western lifestyle, family history (FH), and genetic factors.^2^

About 5% of CRC are considered hereditary due to high-penetrant pathogenic germline variants (PGV) in cancer predisposing genes. The most frequent monogenic cause is Lynch syndrome (LS), with an estimated carrier frequency in the general population of up to 1:300.^3^^,^^4^ It is characterized by an autosomal dominant inherited defect in mismatch repair (MMR) genes (*MLH1*, *MSH2*, *MSH6*, *PMS2*) or *EPCAM* deletions leading to MSH2 silencing. Colorectal cancer lifetime risk in LS varies substantially by gene, sex, and ancestry, with reported cumulative incidences at age 75 ranging from 10% to more than 50%.^5^^,^^6^ LS carriers are prone to early-onset CRC, multiple primary tumors, and other LS-associated malignancies such as endometrial or ovarian cancer. As with other hereditary cancer syndromes with incomplete penetrance, identifying modifiers of cancer risk remains a major challenge.^7^ Beyond LS, up to 25% of CRC cases show familial aggregation or early-onset disease, and yet most of these cases remain genetically unexplained, suggesting a multifactorial or polygenic etiology.^8-10^

Genome-wide association studies (GWAS) have identified multiple common, low-penetrance CRC risk variants.^11^^,^^12^ Although each variant confers only a small individual effect, their combined impact, summarized as a polygenic risk score (PRS), can substantially modify CRC risk. Individuals in the highest PRS percentiles show a 2- to 7-fold increased risk in the general population.^13-17^ Interestingly, PRS has been shown to act independently of environmental factors, FH, and PGV in hereditary CRC genes.^2^^,^^18-21^

However, the influence of PRS across different CRC risk groups remains debated. Population-based studies from the UK Biobank (UKBB) suggest a strong modifying effect of PRS in LS carriers,^13^^,^^18^ whereas analyses from clinical-based LS registries report little or no association.^22^^,^^23^ The PRS appears particularly relevant in unexplained early-onset and familial CRC, where individuals in the highest PRS strata demonstrate markedly increased risk.^9^^,^^24^

The objective of this study was to assess the impact of a validated PRS comprising 93 CRC-associated variants on CRC risk across distinct risk groups.

## Material and methods

### Study population

Data were obtained from the prospective registry of the German Consortium for Familial Intestinal Cancer (GC-FIC). Participants provided written informed consent, and the registry was approved by the ethics committees of the participating institutions. Five university centers enrolled families suspected of LS based on Amsterdam I/II or revised Bethesda criteria since 1990.^25^^,^^26^ Tumor tissue (cancer or adenoma) from index patients was examined for MMR deficiency (dMMR) using immunohistochemistry and/or microsatellite analysis. In the case of dMMR or unavailable tissue, germline testing of *MLH1, MSH2, MSH6, PMS2*, and *EPCAM* was performed using leukocyte DNA. Details about the diagnostic procedure have been described elsewhere.^27^ Data quality was ensured through centralized automated checks for completeness, plausibility, and consistency.

Patients were eligible if they had unexplained CRC fulfilling at least one Bethesda criterion or carried a (likely) PGV in an LS-related gene, regardless of the phenotype. Based on medical history, FH, and germline status, participants were assigned to 3 clinically predefined high-risk groups (Table 1): (1) early-onset sporadic CRC (EOS-CRC), (2) familial CRC (F-CRC) including several subgroups, and (3) confirmed Lynch syndrome (LS). Lynch syndrome index patients were defined as CRC patients with a (likely) PGV (class 4/5) in a MMR gene. Relatives with the same PGV without CRC, including individuals with other LS-associated cancers, were classified as non-CRC LS carriers. From each LS family, only 1 patient with CRC was selected as index, either based on FH information or genotype-derived kinship coefficients. Another risk group (4) encompasses the EOS-CRC and F-CRC patients with conclusive molecular results of the tumor analysis according to the MMR status (deficient/proficient) and/or microsatellite status (instable/stable).

**Table 1.** Summary table.

Patient subgroups	**Definition of subgroups**	CRC cases	Non-CRC cases
Total no. of cases		2198	3541
Sex			
Male		1189	1645
Female		1009	1896
Age, mean (SD)			
GC-FIC		50.5 (13.1)	43.5 (15.1)
UK Biobank		77.2 (1.9)	–
Population controls		–	74.2 (8.0)
Subgroups			
Population controls	Individuals from the Heinz Nixdorf Recall cohort study	0	3119
Sporadic CRC	Patients without documented CRC cases in first and second-degree relatives	1299	0
EOS-CRC	Age of onset <50 years	518	0
LOS-CRC	Age of onset ≥75 years (individuals UKBB)	781	0
Familial CRC	Patients diagnosed with CRC and with documented 1 or more first or second-degree family members with CRC	220	0
F-CRC-1	Age of onset <50 years, relative with CRC	57	0
F-CRC-2	Age of onset ≥50 years, at least 2 relatives with CRC, including 1 first-degree relative with age of onset <50 years	60	0
F-CRC-3	Age of onset ≥50 years, at least 2 relatives with CRC, no first-degree relative with age of onset <50 years	103	0
Lynch syndrome	CRC-affected LS index patients and non-CRC relatives of LS index patients with a class 4/5 PGV in *MLH1, MSH2, MSH6*, or *PMS2*	679	422
*MLH1*	Carrier of PGV in *MLH1*	283	139
*MSH2*	Carrier of PGV in *MSH2*	305	192
*MSH6*	Carrier of PGV in *MSH6*	64	70
*PMS2*	Carrier of PGV in *PMS2*	27	21

Abbreviations: GC-FIC = German Consortium for Familial Intestinal Cancer; PGV = pathogenic germline variant; UKBB = UK Biobank; EOS = early-onset sporadic; LOS = late-onset sporadic; F-CRC = familial colorectal cancer.

Healthy population controls were drawn from the German population-based Heinz Nixdorf RECALL (HNR) cohort study, comprising individuals aged 45 to 75 years from the Ruhr area, as described previously.^28^ Individuals with CRC were excluded; the PGV status of the MMR genes was unknown. Additionally, individuals with late-onset sporadic CRC (LOS-CRC; age at diagnosis ≥75 years) of White British ancestry were included from the UK Biobank (UKBB) under application number 81202.^29^ CRC individuals were identified via ICD-9 and ICD-10 codes.

### Genotyping

The leukocyte-derived DNA was genotyped with the Illumina Infinium Global Screening Array (GSA) v3.0 (Illumina, Inc., San Diego, CA, USA) using a semiautomated protocol. All laboratory procedures were performed in accordance with the manufacturer’s instructions. Illumina raw intensity files were uploaded together with the Illumina GSA manifest and cluster file into the GenomeStudio software, and genotypes were subsequently exported to PLINK format. Quality control was performed using PLINK to ensure a minor allele frequency of at least 1% and a call rate of at least 99% per variant.

Non-European descendant individuals were excluded from the analysis. To assess the ethnicity, the samples were compared with 1k genome samples. Classification in the different ethnicity groups was performed by selecting ancestry-informative marker single-nucleotide polymorphisms (SNPs) and using a principal component analysis (PCA) approach.

### Imputation

An established PRS for CRC with 95 common SNPs was considered.^11^ Eighteen of the 95 variants of interest were included in the Illumina GSA v3.0. The variants not directly genotyped were imputed with a local pipeline that is comparable to the Michigan Imputation Server and based on the bioinformatics tools bcftools, minimac, and vcftools using GRCh37 as the reference genome (1000 Genomes, phase 3, v5), as previously described.^22^ Missing variants and variants with an imputation quality (r2) lower than 0.3 were not included in the final PRS, which resulted in the exclusion of rs6058093 (effect size 0.045, risk allele frequency 0.4942) and rs755229494 (effect size 0.6286, risk allele frequency 0.0011). Due to either a small effect size or a very low risk allele frequency, we expect only a minor impact of the exclusion of those variants. Imputation results of the remaining 93 variants are included in Table S1.

### PRS calculation

For each participant, a PRS was calculated using PLINK 2.0,^30^^,^^31^ based on 93 quality-controlled CRC-associated variants and their reported effect sizes.^11^ Participants were categorized according to PRS percentiles derived from CRC-free population controls: low PRS (<20th percentile), intermediate PRS (20th-80th percentile; reference), and high PRS (>80th percentile). PRS calculation in UKBB followed the same protocol and was based on the same variants. The PRS distribution in CRC-free individuals and EOS-CRC cases in UKBB was comparable to the population controls and EOS-CRC cases in the present study, respectively.

### Statistical analysis

Differences in PRS between risk groups were assessed using a 1-sided 2-sample *t*-test, testing whether mean PRS was higher in CRC cases than in controls. When related individuals were included, a linear mixed model with family ID as a random effect was applied.

Associations between PRS and CRC risk were evaluated using sex-adjusted logistic regressions, with CRC status as the outcome and PRS modeled either continuously or categorically. Intermediate PRS served as the reference category, and mixed models were used when familial relationships were present. Results from continuous PRS are reported as adjusted odds ratios (OR) per standard deviation (SD).

Cumulative CRC incidence was analyzed using Cox proportional hazard models with age at diagnosis (cases) or age at last visit (controls/non-CRC LS carriers) as event times, adjusted for sex. Frailty models including family ID were fitted when applicable. Estimated hazard ratios (HR) are presented per standard deviation (SD) for continuous PRS.

All *P* values were corrected for multiple testing using the false discovery rate (FDR^32^), with statistical significance defined as α = .05 after correction.

Due to an existing age gap between CRC risk groups and population controls (see Table 1), as a sensitivity analysis, population controls were matched 1:1 to CRC risk groups using propensity scores and age at last contact. Results are shown in Table S2.

## Results

### Characterization of patients

**Figure 1.**
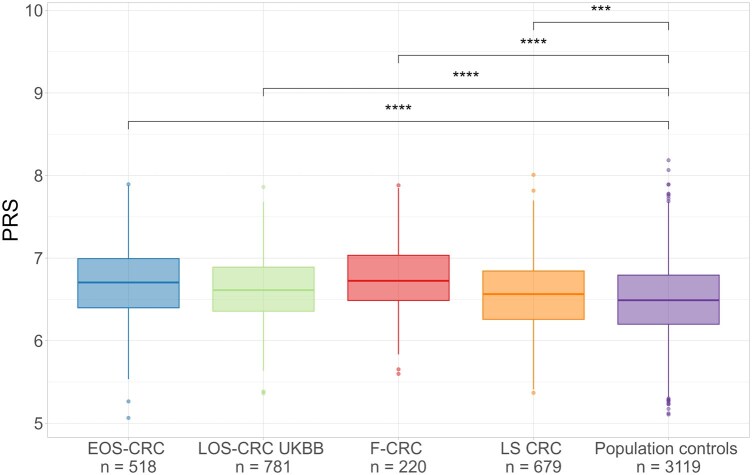
Boxplots displaying the distribution of PRS values in the different subgroups. Only unrelated individuals and individuals diagnosed with colorectal cancer (CRC) (except for population controls) are shown. A 2-sample *t* test was performed with the 1-sided alternative of CRC cases showing a higher mean PRS than population controls. Asterisks refer to FDR-corrected *P* values: ns: nonsignificant, *P < .05, **P < .01, ***P < .001, ****P < .0001. Abbreviations: EOS-CRC = early-onset sporadic CRC; LOS-CRC UKBB = late-onset sporadic CRC from UK Biobank; F-CRC = familial CRC; LS-CRC = Lynch syndrome associated CRC.

Overall, 5739 individuals were included: 1839 GC-FIC probands, 3119 CRC-free German population controls (HNR), and 781 were LOS-CRC cases from the UKBB (Table 1). Polygenic risk score distributions across unrelated individuals are shown in Figure 1. All CRC risk groups had significantly higher PRS than population controls (all *P* < .02). Mean PRS was highest in EOS-CRC and F-CRC, and lowest in LOS-CRC.

### Early- and late-onset sporadic CRC

**Figure 2.**
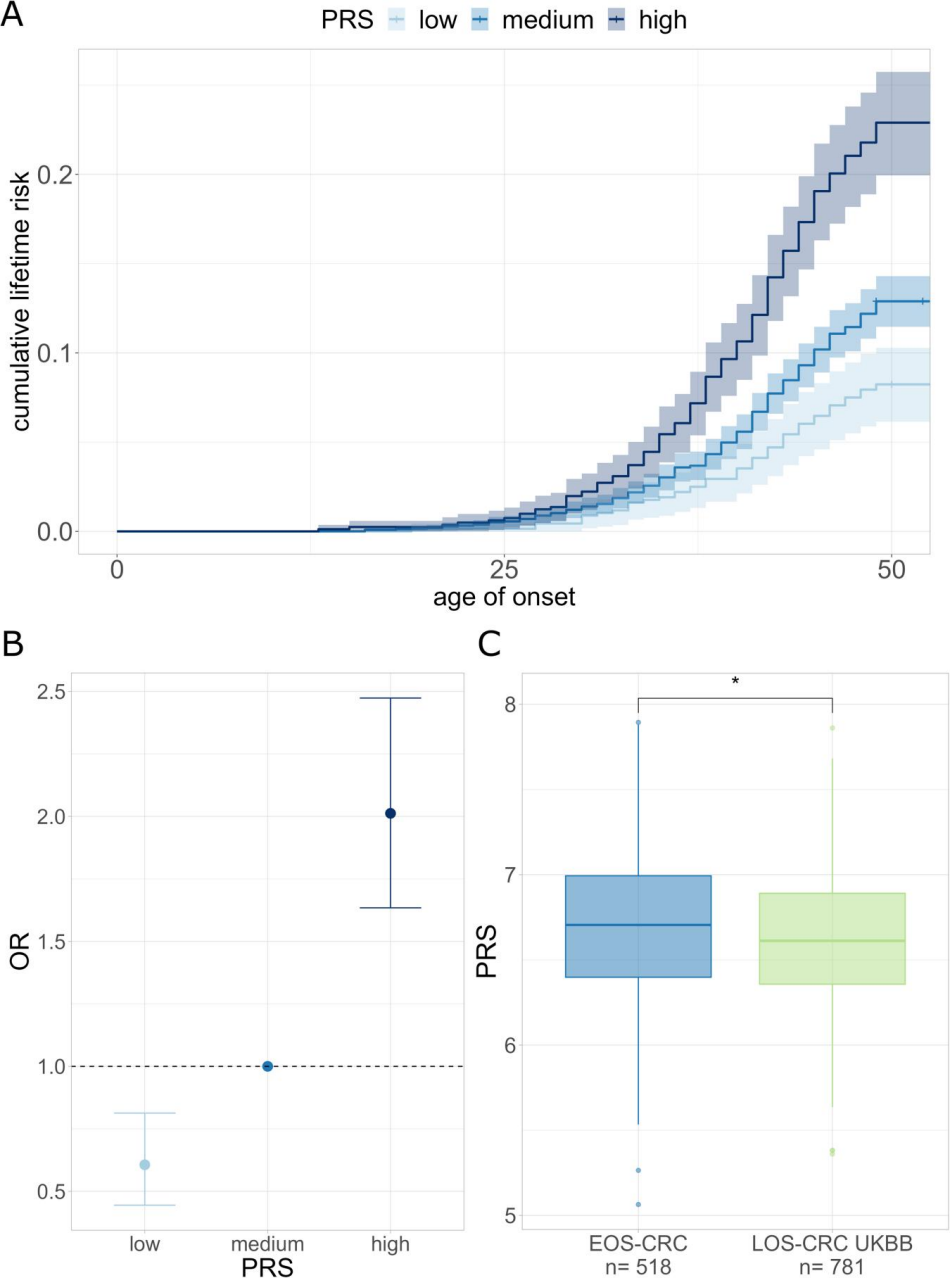
Analysis of sporadic colorectal cancer (CRC) cases. **(A)** Kaplan-Meier curves derived from early-onset sporadic (EOS)-CRC cases and population controls. Individuals are stratified based on their PRS (low: lowest 20% PRS, high: highest 20% PRS, medium: in between). Shaded areas along the Kaplan-Meier curves indicate 95% confidence intervals. **(B)** Estimated odds ratio of PRS categories via logistic regression on EOS-CRC cases and population controls. **(C)** Boxplots comparing EOS-CRC cases and late-onset sporadic (LOS)-CRC cases. A 1-sided *t* test was performed. Asterisks refer to FDR-corrected *P* values: ns = nonsignificant, *P < .05, **P < .01, ***P < .001, ****P < .0001.

The EOS-CRC subgroup included 518 unrelated individuals. Comparing this group with the non-CRC population controls (*n* = 3119), a higher PRS was associated with increased CRC risk (OR [per SD] = 1.55, 95% CI = 1.41 to 1.71) and cumulative incidence (HR [per SD] = 1.49, 95% CI = 1.37 to 1.63). Compared with intermediate PRS, the 20% of individuals within the high PRS group had a 2-fold increased CRC risk (OR = 2.01, 95% CI = 1.63 to 2.47), whereas individuals within the low PRS group showed a significantly reduced risk of developing CRC (OR = 0.61, 95% CI = 0.44 to 0.81) (Figure 2). Table 2 summarizes the estimated cumulative incidence of CRC until the age of 50 years, which differed between roughly 24% in the high PRS group, around 13% the medium PRS group, and around 8% in the low PRS group. Individuals with a PRS in the top 10% of the PRS distribution showed an even higher cumulative incidence (Table 2). Results were consistent in very early-onset cases (<40 years; Table S3). EOS-CRC cases had significantly higher PRS than LOS-CRC cases (*P *< 0.02; Figure 2C).

**Table 2.** Estimated cumulative incidence of CRC in the EOS and F-CRC group using different PRS categories.

Risk group	PRS category	Cumulative incidence (95% CI)	
		Male	Female
EOS-CRC, standard PRS categories	Low (lower 20%)	9% (6% to 11%)	8% (6% to 10%)
	Medium (20 to 80%)	13% (12% to 15%)	12% (11% to 14%)
	High (upper 20%)	24% (20% to 27%)	22% (19% to 25%)
EOS-CRC, alternative PRS categories	Low (lower 10%)	10% (7% to 14%)	9% (6% to 13%)
	Medium (10 to 90%)	14% (12% to 15%)	13% (11% to 14%)
	High (upper 10%)	27% (22% to 32%)	25% (20% to 29%)
F-CRC, standard PRS categories	Low (lower 20%)	3% (1% to 4%)	3% (1% to 4%)
	Medium (20 to 80%)	6% (5% to 8%)	6% (4% to 7%)
	High (upper 20%)	14% (10% to 17%)	12% (9% to 15%)

### Familial CRC

**Figure 3.**
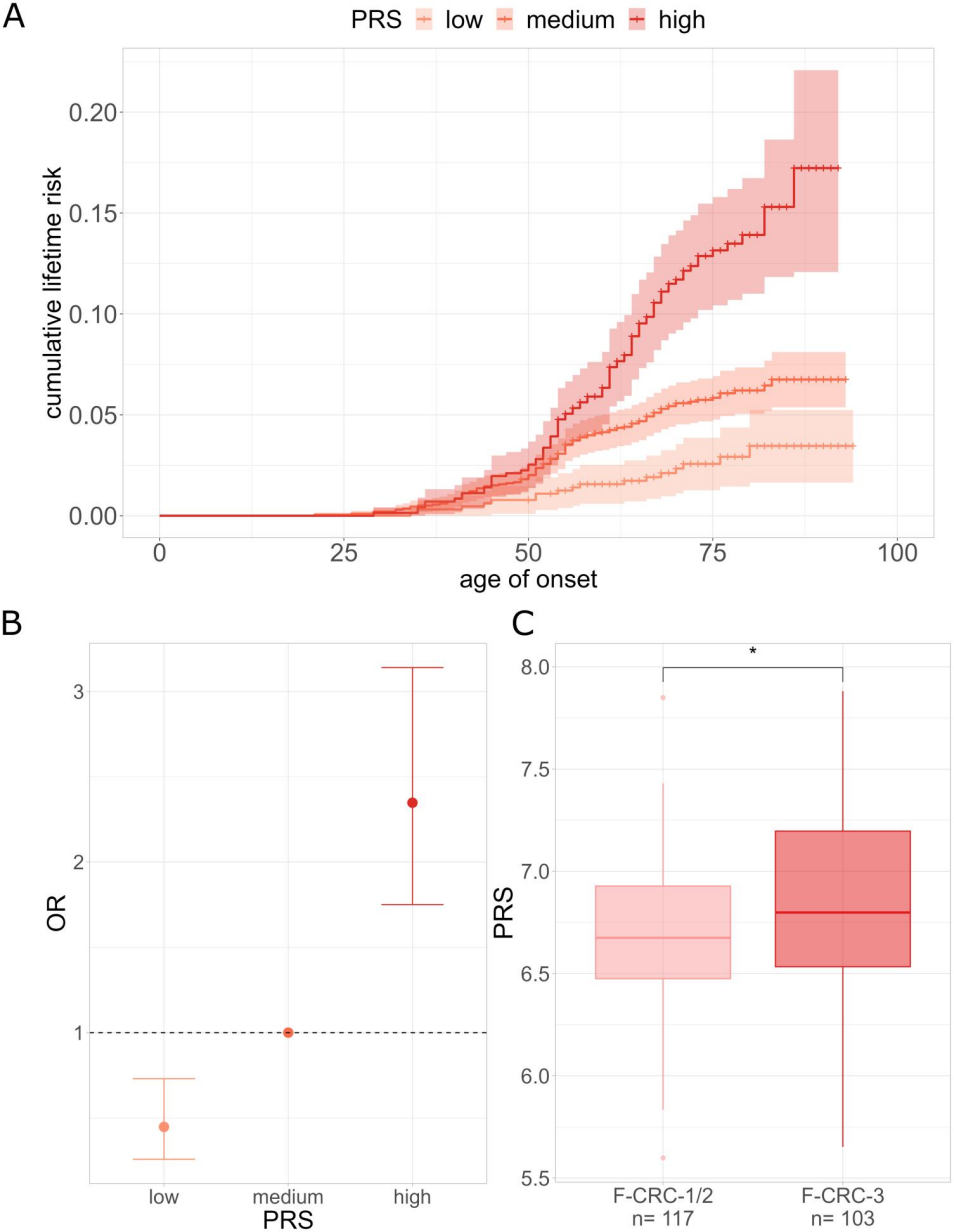
Analysis of familial colorectal cancer cases (F-CRC). **(A)** Kaplan-Meier curves derived from F-CRC cases and population controls. Individuals are stratified based on their PRS (low: lowest 20% PRS, high: highest 20% PRS, medium: in between). Shaded areas along the Kaplan-Meier curves indicate 95% confidence intervals. **(B)** Estimated odds ratio of PRS categories via logistic regression on F-CRC cases and population controls. **(C)** Boxplots comparing 2 subgroups of F-CRC: F-CRC-1/F-CRC-2 combined and F-CRC-3. A 2-sided *t*-test was performed. Asterisks refer to FDR-corrected *P* values: ns = nonsignificant, *P < .05, **P < .01, ***P < .001, ****P < .0001.

Among unrelated F-CRC cases and non-CRC population controls (*n* = 220 F-CRC cases, *n* = 3119 controls), a higher PRS was associated with increased CRC risk (OR [per SD] = 1.80, 95% CI = 1.56 to 2.07) as well as cumulative incidence (HR [per SD] = 1.75, 95% CI = 1.53 to 1.99). By age 75, CRC incidence increased from roughly 3% in the low PRS group to around 13% in the high PRS group (Figure 3A, Table 2). The 20% of individuals within the high PRS group showed a more than 2-fold increased CRC incidence compared with individuals within the intermediate PRS group (OR = 2.35, 95% CI = 1.75 to 3.14, HR = 2.24, 95% CI = 1.70 to 2.96; Figure 3B), whereas individuals within the low PRS group showed a decreased CRC incidence (OR = 0.45, 95% CI = 0.26 to 0.73, HR = 0.45, 95% CI = 0.27 to 0.76).

Subsequently, 3 different F-CRC subgroups, based on age at onset and affected relatives, were analyzed (Table 1). The subgroups F-CRC-1 (CRC <50 years, relative with CRC) and F-CRC-2 (CRC ≥50 years, first-degree relative with CRC <50 years) demonstrated the same direction of the effect, although the results did partly not reach statistical significance (likely due to the small sample size). The strongest effect was observed in the subgroup with multiple late-onset affected individuals (F-CRC-3; age of onset ≥50 years, at least 2 first- or second-degree relatives with CRC, no first-degree relative with CRC <50 years), who showed a 3-fold increased CRC risk in the high PRS (OR [per SD] = 3.1, 95% CI = 2.1 to 4.8; Table S3). In addition, we found F-CRC-3 cases to have a significantly increased PRS compared with F-CRC-1 and F-CRC-2 cases (Figure 3C).

### Lynch syndrome

We considered 2 LS subgroups and compared them with the non-CRC individuals from the HNR cohort (“population controls”; *n* = 3119): (1) individuals diagnosed with CRC and a PGV in a LS-associated gene (“LS CRC individuals”; *n* = 679); (2) individuals with a PGV without CRC (“non-CRC LS carriers”; *n* = 422). The 2 LS cohorts comprised related individuals; thus, mixed models were used for the statistical analysis.

**Figure 4.**
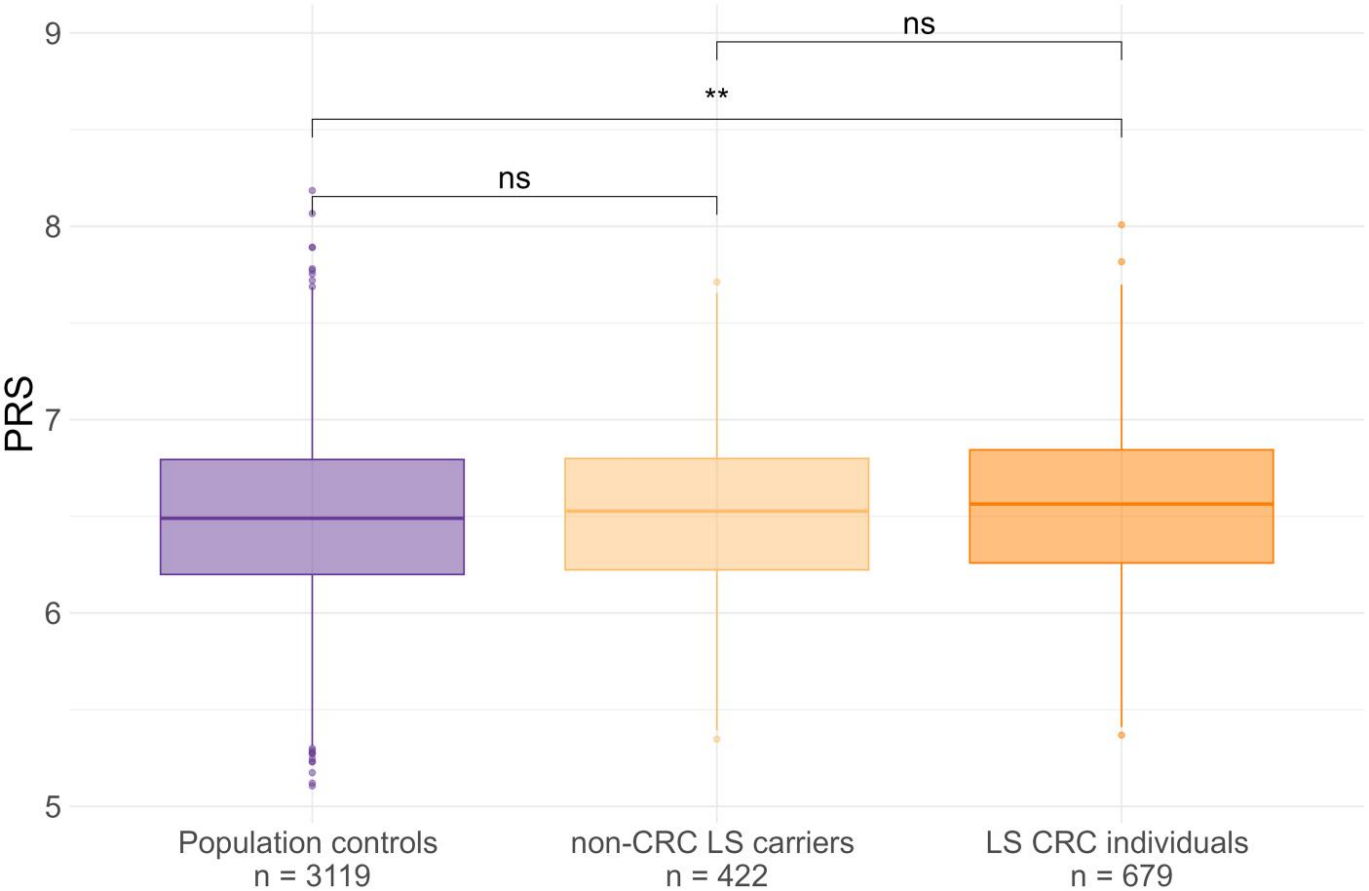
Boxplots of PRS of population controls, Lynch syndrome carriers that have not been diagnosed with CRC (non-CRC LS carriers), and Lynch syndrome carriers that have been diagnosed with CRC (LS CRC individuals). PRS were compared via linear mixed models with group as covariate and family ID as random effect. Asterisks refer to FDR-corrected *P* values: ns = nonsignificant, *P < .05, **P < .01, ***P < .001, ****P < .0001.

Lynch syndrome CRC individuals showed a significantly increased PRS compared with population controls (*P* < .002). However, we did not observe a statistically significant difference in PRS between non-CRC LS carriers and population controls (95% CI = −0.03 to 0.07) and between LS CRC individuals and non-CRC LS carriers (95% CI = −0.01 to 0.09) (Figure 4).

To take the different CRC penetrance of the MMR genes into account, we divided the LS individuals into 2 groups: those that showed a PGV in the high penetrant genes *MLH1* (*n* = 283 LS CRC individuals, *n* = 139 non-CRC LS carriers) and *MSH2* (*n* = 305 LS CRC individuals, *n* = 192 non-CRC LS carriers) and those with a PGV in 1 of the moderate to low penetrant genes *MSH6* (*n* = 64 LS CRC individuals, *n* = 70 non-CRC LS carriers) and *PMS2* (*n* = 27 LS CRC individuals, *n* = 21 non-CRC LS carriers). We did not find a significantly increased PRS in LS CRC individuals compared with non-CRC LS carriers in any of these groups (Figure S1). Compared with population controls, we found a significantly increased PRS in LS CRC individuals with a PGV in *MSH2* (*P *< .01) but not in *MLH1* (*P *= .15), *MSH6* (*P *= .35), or PMS2 (*P *= .70).

### Tumor specification

**Figure 5.**
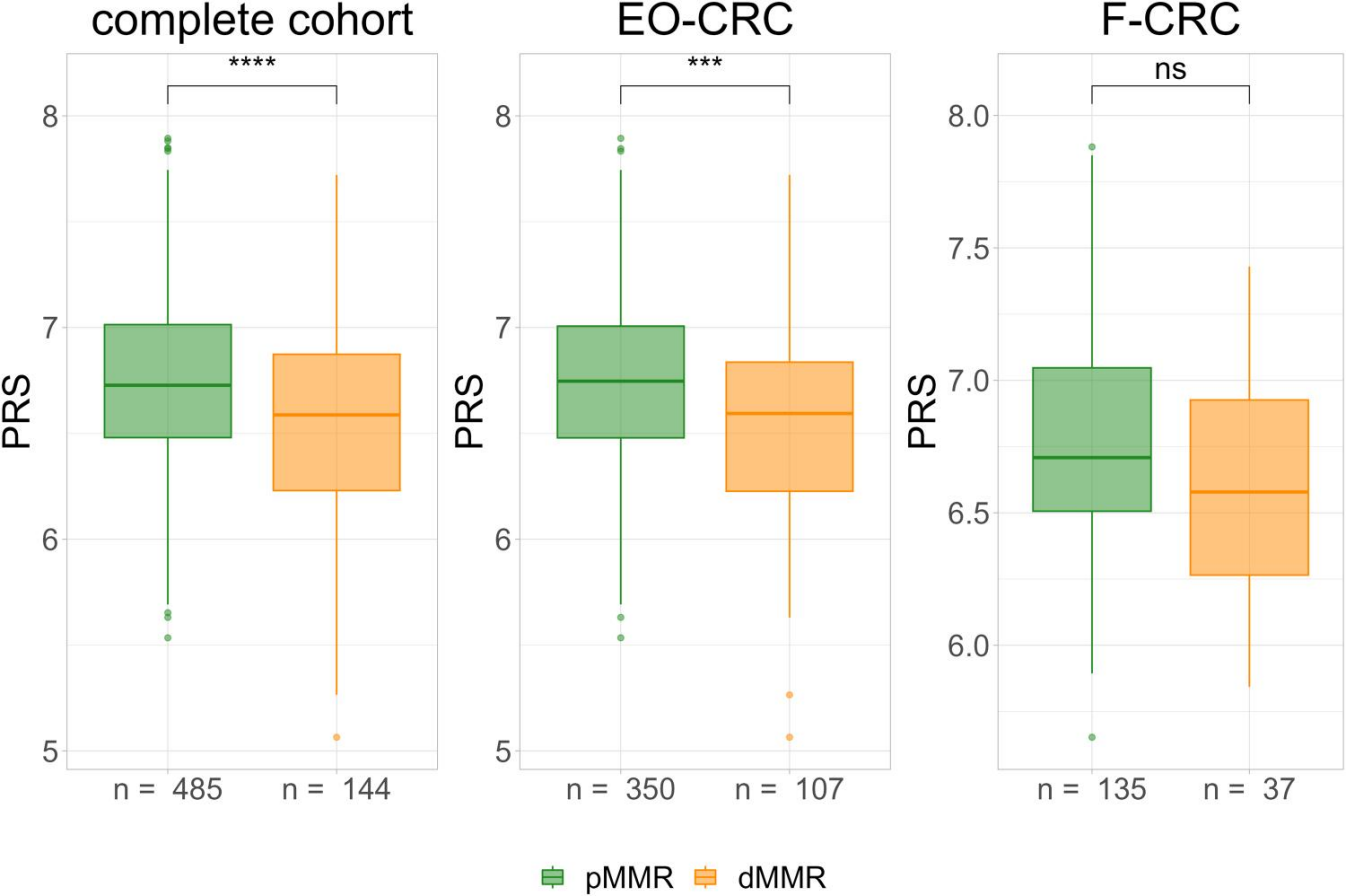
Boxplots comparing PRS of non-LS individuals with normal tumor tissue (pMMR) and with dMMR/MSI-H results. The complete non-LS cohort is split into early-onset sporadic CRC cases (EOS-CRC) and familial CRC cases (F-CRC). Comparisons were performed via 2-sided *t* tests. Asterisks refer to FDR-corrected *P* values: ns = nonsignificant, *P < .05, **P < .01, ***P <0.001, ****P < .0001.

We further divided the genetically unexplained CRC patients of the different, non-LS risk groups (EOS-CRC and F-CRC patients) according to the available and conclusive molecular results of the tumor analysis into those with a LS-convenient result, ie, a deficient MMR status (dMMR) and/or high microsatellite instability (MSI-H; *n* = 144) and those with normal results of tumor examination, ie, a proficient MMR status (pMMR) and/or microsatellite stability (MSS) (*n* = 485). Related individuals (*n* = 4) were excluded from the analysis. We found a significantly lower mean PRS in the individuals with MSI/dMMR compared with MSS/pMMR (*t* test, *P *< .001). These results were consistent when the separate subgroups (EOS-CRC: *n* = 107 dMMR, *n* = 350 pMMR, F-CRC cases: *n* = 37 dMMR, *n* = 135 pMMR; Figure 5) were considered. It is noteworthy that we found no significant difference in PRS in individuals with unexplained dMMR/MSI CRC compared with LS CRC individuals (*P* = .82).

In summary, the PRS was significantly increased in all CRC groups compared with population controls. Being in the high PRS category doubled CRC risk in EOS-CRC and F-CRC corresponding to cumulative incidences before 50 and 75 years of 24% and 13%, respectively, whereas CRC risk in the low PRS group is roughly halved. Polygenic risk score was significantly higher in EOS-CRC than LOS-CRC. The strongest effect in F-CRC with a 3-fold increased risk in the high PRS category was observed in the subgroup with multiple late-onset affected individuals. In LS, mean PRS in non-CRC carriers lay between CRC-LS and population controls. Finally, non-LS individuals with MSI/dMMR tumors showed significantly lower PRS than those with pMMR/MSS tumors, and no difference compared with LS CRC individuals.

## Discussion

Established risk factors for CRC include a Western lifestyle, a more severe clinical presentation such as early-onset disease or familial clustering of CRC, and monogenic forms of hereditary CRC, particularly Lynch syndrome.^21^^,^^33^ Recent studies demonstrated that the risk of CRC—as well as of several other common cancers—is also modified considerably by the polygenic background, defined as a PRS based on disease-associated SNPs, both in terms of age at onset and cumulative lifetime risks.^15^^,^^16^^,^^34-37^ Individuals in the lowest polygenic risk strata (<20%) exhibit, on average, an approximately 4-fold reduction in CRC risk, with those in the lowest 1% having up to a 70% lower risk than the mid-quintile.^35^ Conversely, individuals in the top 20% and top 1% have roughly 2-fold and 4-fold higher risks, respectively. Hence, individuals with a very high PRS may have CRC risk levels comparable to carriers of monogenic variants but with a low PRS.^13^^,^^38^ Similar PRS-dependent patterns have recently been reported for colorectal adenomas and post-colonoscopy CRCs.^38^

Consistent with these observations, CRC-associated risk alleles tend to accumulate in unexplained familial and early-onset CRC cases.^9^^,^^24^^,^^39^ Moreover, PRS has been shown to modulate risk even among individuals with LS.^18^^,^^22^ However, the impact of the PRS strongly depends on study design, recruitment, and analytical methods.^17^^,^^18^^,^^22^^,^^23^

To explore how common genetic susceptibility contributes to CRC risk, we analyzed data from the GC-FIC cohort including unexplained early-onset sporadic CRC (EOS-CRC), unexplained familial CRC (F-CRC), and LS individuals (affected and unaffected), compared with population controls and late-onset sporadic CRC (LOS-CRC) cases. We applied a well-established PRS model for CRC, composed of 95 SNPs, that was used in several CRC PRS studies. In previous studies, we and others analyzed further PRS models (140 SNPs, 50 SNPs) that were replicated in a meta-analyzed GWAS after excluding UKBB samples and could demonstrate that all 3 PRS models had comparable performance.^18^

All CRC risk groups showed significantly higher mean PRS than controls, with the highest values in early-onset and familial cases, consistent with previous findings.^40^ The elevated PRS in the EOS-CRC relative to LOS-CRC supports its predictive validity. Although LOS-CRC showed the smallest increase, the PRS is still significantly higher compared with population controls.

Across groups, a high PRS (>80th percentile) conferred approximately double CRC risk, whereas a low PRS (<20th percentile) substantially reduced it. Depending on PRS category, EOS-CRC incidence by age 50 ranged from 8% to 24%, and F-CRC incidence by age 75 ranged from 3% to 13%, consistent with prior reports.^38^ In Germany, a preventive screening colonoscopy is recommended for the general population by age 50, when the population cumulative CRC incidence is estimated to be around 0.55% and the prospective absolute 10-year risk around 1%.^41^ This threshold is reached by F-CRC individuals with high PRS around age 34 (Figure 3A), suggesting that screening might be advanced by approximately 15 years for this subgroup.^38^^,^^42^^,^^43^

Interestingly, the strongest polygenic effect, corresponding to a 3-fold increased risk, was seen in families with a clustering of late-onset CRC (F-CRC-3; Figure 3). Due to the positive FH, it could be expected that the polygenic impact is (much) higher compared with LOS-CRC (Figure 1). This observation supports the hypothesis that a clustering of late-onset cancers arises predominantly through low penetrant genetic and nongenetic factors rather than highly penetrant variants, whereas in early-onset cases (with or without a FH), additional (moderate) penetrant genetic risk factors, not captured by the PRS, might be present.

Although EOS-CRCs are often suspected to be monogenic, only 16% to 35% carry a PGV in established hereditary CRC genes, compared with 2% to 5% in overall CRC.^44-49^ Thus, a certain fraction of unexplained EOS-CRC cases likely result from the accumulation of low-penetrant SNPs rather than rare, high-impact variants.

Stratification of the genetically unexplained patients with clinically suspected LS (EOS-CRC and F-CRC cases) revealed significantly higher PRS in those with pMMR/MSS tumors than in MSI/dMMR cases, suggesting a stronger polygenic contribution when no monogenic cause is evident. Polygenic risk score did not differ significantly between MSI/dMMR and confirmed LS CRCs, implying that some unexplained MSI/dMMR tumors may represent undetected LS. This interpretation is consistent with their similar risk of metachronous neoplasia.^50^

No significant PRS differences were detected between non-CRC LS carriers and population controls, nor between non-CRC LS carriers and LS CRC individuals. This is in line with previous studies, although the mean values followed the expected gradients (LS CRC > non-CRC LS carriers > population controls).

The nonsignificant PRS differences between CRC-LS and non-CRC-LS might be influenced by various factors. Recent analyses indicate that PRS modifies CRC risk in LS depending on the affected gene, with the strongest modulation seen in moderate/low-penetrant *MSH6* and *PMS2*.^18^ As in other clinically ascertained LS cohorts,^22^^,^^23^ and in contrast with population-based cohorts,^18^ most carriers in this study (87%) harbored PGVs in the high-penetrant genes *MLH1* or *MSH2*, which are likely to be less influenced by the genetic background. This overrepresentation of *MLH1*/*MSH2* likely explains why only a modest PRS increase was observed for *MSH2*-associated CRCs. Furthermore, and again in contrast to population-based studies, non-CRC LS carriers were mainly relatives of index patients, and thus the shared polygenic background likely explains the intermediate PRS values; larger sample sizes may yield significance. The results are further modified by the different composition of cohorts with much more non-CRC-LS individuals compared with CRC-LS individuals in population-based cohorts as in clinically ascertained cohorts. It was also speculated that LS-specific SNPs might exist, so that the CRC-associated SNPs identified in recent CRC GWAS do not fully represent the relevant polygenic background. Consequently, the design and recruitment strategy (clinically based vs population-based) strongly influences the results, interpretation, and conclusions of PRS studies.

All 3 high-risk groups (EOS-CRC, F-CRC, LS) are recruited based on clinical criteria (revised Bethesda criteria), so that an accumulation and heterogeneous distribution of different genetic and nongenetic risk factors compared with population controls (and LOS-CRC) can be expected, which may bias effect estimates. The F-CRC cohort and most LS cases have a positive FH as an additional risk factor, which resulted in higher overall effect estimates across all PRS categories according to previous data.^18^ In EOS-CRC, instead, genetic effects are typically stronger,^24^ and thus a pronounced contribution of moderate penetrant alterations not covered by PRS can be hypothesized. On the other hand, the relative contribution of these risk factors (PRS, FH, lifestyle) in LS is supposed to be lower given the PGV as the strongest CRC risk factor. Although LOS-CRC cases can be assumed to have in general a low contribution of major risk factors, this is expected to be different in familial late-onset (subgroup F-CRC-3), where in the absence of known moderate and high penetrant genetic factors in particular low-penetrant genetic factors are dominantly contributing to CRC risk. In F-CRC in general, the PRS might be the strongest risk factor, since in patients with low PRS, CRC risk is lower even with a positive CRC FH.

This study has limitations, including small subgroup sizes and incomplete PGV data for population controls. However, given the rarity of PGV in the general population and the exclusion of CRC cases among the controls, this should not have affected the results substantially. When interpreting the data, it is important to keep in mind that the vast majority of non-CRC LS carriers, ie, mainly healthy LS carriers, are related to a CRC-affected LS patient. Hence, the results are relevant for so far not CRC-affected carrier relatives of affected LS patients, but not for healthy carriers with unsuspicious FH, which are identified by population screening or in the context of an incidental/secondary genetic finding and where the PRS and CRC risk might be lower.

Population controls from the HNR study were considerably older than individuals from the CRC risk groups. Although this reduces the risk of future CRC cases in the population controls, a homogeneous distribution of age and year of birth would increase comparability. However, our sensitivity analysis using age matching showed the robustness of our results (Table S2). In this as in other PRS studies, hereditary CRC was restricted to LS. To explore the impact of PRS in other, rare monogenic CRC types, large collaborative international efforts are needed to ascertain meaningful cohorts.

We did not include other ancestral groups due to the ethnic composition of the GC-FIC registry. The GWAS data used for current PRS were generated and validated in individuals of European ancestry, limiting the applicability for other ethnicities. However, different approaches are being developed to overcome this restriction.^18^^,^^51^

In summary, to our knowledge, this is the first analysis of SNP-based polygenic risk score across these diverse CRC-risk groups. The PRS exerted the strongest effect in unexplained early-onset and familial cases, whereas its influence in LS depended on MMR gene penetrance and study design. Since CRC risk stratification based on age, FH, and monogenic conditions is already recommended in several guidelines and partly established in medical care, the integration of a PRS as an additional risk predictor in the stratification workflow should be viable. However, although the amount of current data provides a straightforward basis for designing PRS-based population CRC screening programs, some substantial barriers have yet to be solved. A roadmap may include further research to better estimate absolute risks and to propose risk-adopted surveillance recommendations based on the average CRC risk level currently accepted for routine CRC public surveillance (preventive activities at different levels of risk). In addition, regional or national prospective clinical pilot trials are needed to address the acceptance, feasibility, clinical utility, and cost-effectiveness of PRS-based risk programs. Ultimately, integrating environmental, monogenic, and polygenic factors into comprehensive models could advance individualized, risk-adapted CRC prevention strategies.^52-55^ However, although comprehensive prediction tools including all major risk factors are still challenging, models with only PRS (and FH) might be a feasible first step toward a more accurate CRC risk prediction.

## Supplementary Material

pkag041_Supplementary_Data

## Data Availability

The data that support the findings of this study are available upon reasonable request. Access may be subject to data protection regulations and ethical considerations.
