# Supplementary material for: Effect of a polygenic risk score in patients with late-onset, early-onset, familial, or hereditary colorectal cancer
Source: JNCI Cancer Spectr. 2026 Apr 11;10(3):pkag041. doi: 10.1093/jncics/pkag041 (PMC13263116; doi:10.1093/jncics/pkag041)
Supplement: pkag041_Supplementary_Data [file pkag041_supplementary_data.zip › Klinkhammer_JNCICS_R1_supplementary_material.docx]

**Supplementary Material**

**Table S1** Imputation results of CRC-associated risk variants

**Table S2** Summary table of conducted analyses and results in age-matched cohorts

**Table S3** Summary table of conducted analyses and results

**
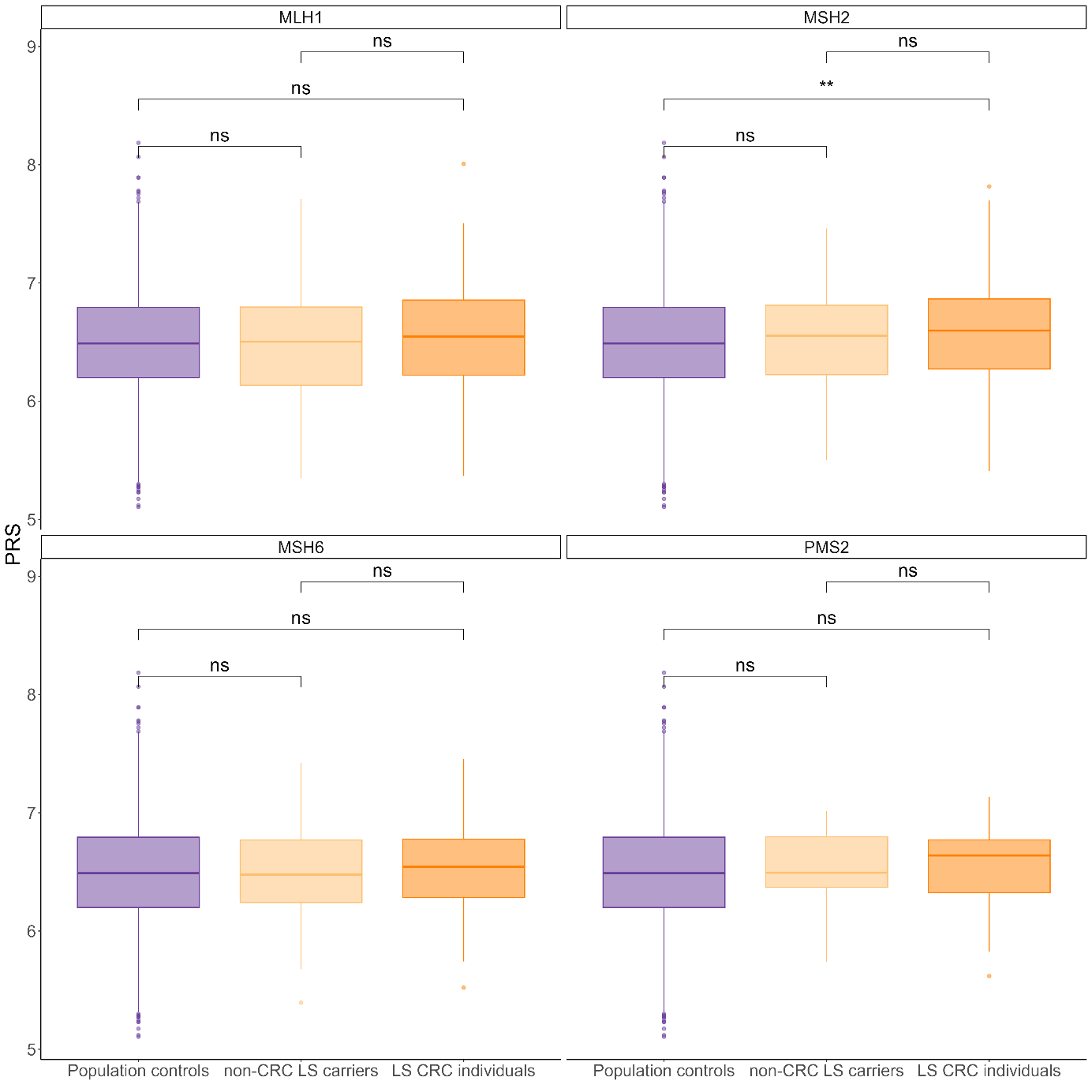
**

**Figure S1** Boxplots of PRS of population controls, Lynch syndrome carriers that have not been diagnosed with CRC (non-CRC LS carriers) and Lynch syndrome carriers that have been diagnosed with CRC (LS CRC individuals) split by affected gene. PRS were compared via linear mixed models with group as covariate and family ID as random effect. Asterisks refer to FDR-corrected p-values: ns: non-significant, *: p<0.05, **: p<0.01, ***: p<0.001, ****: p<0.0001.
